# Chronic sleep loss disrupts rhythmic gene expression in *Drosophila*

**DOI:** 10.3389/fphys.2022.1048751

**Published:** 2022-11-18

**Authors:** Zikun Wang, Samantha Lincoln, Andrew D. Nguyen, Wanhe Li, Michael W. Young

**Affiliations:** ^1^ Laboratory of Genetics, The Rockefeller University, New York, NY, United States; ^2^ Department of Biology, Center for Biological Clocks Research, Texas A&M University, College Station, United States

**Keywords:** chronic sleep loss, rhythmic gene expression, transcriptome profiling, sleep, circadian rhythm

## Abstract

Genome-wide profiling of rhythmic gene expression has offered new avenues for studying the contribution of circadian clock to diverse biological processes. Sleep has been considered one of the most important physiological processes that are regulated by the circadian clock, however, the effects of chronic sleep loss on rhythmic gene expression remain poorly understood. In the present study, we exploited *Drosophila* sleep mutants *insomniac* ^*1*^ (*inc* ^*1*^) and *wide awake* ^*D2*^ (*wake* ^*D2*^) as models for chronic sleep loss. We profiled the transcriptomes of head tissues collected from 4-week-old wild type flies, *inc* ^*1*^ and *wake* ^*D2*^ at timepoints around the clock. Analysis of gene oscillation revealed a substantial loss of rhythmicity in *inc* ^*1*^ and *wake* ^*D2*^ compared to wild type flies, with most of the affected genes common to both mutants. The disruption of gene oscillation was not due to changes in average gene expression levels. We also identified a subset of genes whose loss of rhythmicity was shared among animals with chronic sleep loss and old flies, suggesting a contribution of aging to chronic, sleep-loss-induced disruption of gene oscillation.

## Introduction

Many genes exhibit rhythmic expression patterns and are regulated by an internal timing system, the circadian clock. In animals, the circadian clock, regulated by a transcription-translation feedback loop, controls networks of genes oscillating with ∼24-hour periodicity. These in turn influence rhythmic aspects of animal physiology and behavior, most prominently, sleep (Rijo-Ferreira and Takahashi, 2019).

Chronic disruptions of sleep and circadian rhythm are intertwined processes: Sleep, which is considered to be regulated by both a homeostatic and a circadian process (Borbely, 1982), is directly influenced by the circadian clock. However, sleep deprivation not only changes subsequent depth of sleep, but also alters light responsiveness and phase shifting property of the circadian clock (Challet et al., 2001; Mistlberger et al., 2002; Burgess, 2010; van Diepen et al., 2014; Jha et al., 2017). Only a handful of investigations have asked how sleep affects the circadian clock. These studies have demonstrated correlations between the electrical activity of neurons composing the suprachiasmatic nucleus (SCN), the mammalian central clock, and depth of sleep or sleep pressure (Deboer et al., 2003; Deboer et al., 2007; Schmidt et al., 2009). Sleep pressure was also found to attenuate phase shifts induced by light in mice, hamsters and humans (Challet et al., 2001; Mistlberger et al., 2002; Burgess, 2010). These physiological and behavioral effects of sleep on the circadian clock were mostly examined using sleep restriction, caffeine treatment or acute sleep deprivation experiments. How chronic sleep disruption affects rhythmicity remains poorly documented, particularly, on the level of oscillating gene expression.

In mice and primates, respectively ∼40% and ∼80% of all protein-coding genes exhibit daily rhythmic expression (Zhang et al., 2014; Mure et al., 2018). In *Drosophila*, micro-array and RNA-sequencing methods have identified several hundred transcripts whose oscillating properties are regulated by *period*, the first identified clock gene (Claridge-Chang et al., 2001; McDonald and Rosbash, 2001; Hughes et al., 2012). *Drosophila* has been established as an exceptional model for studying the genetic basis of sleep regulation (Shafer and Keene, 2021)*.* Similar to the mammalian brain, spontaneous sleep, spontaneous wakefulness and sleep deprivation in *Drosophila* all directly modulate gene expression (Cirelli et al., 2005). *Drosophila* sleep mutants can serve as an efficient and inexpensive method to achieve chronic sleep disruption in a large number of animals for profiling rhythmic gene expression. Here we report a substantial loss of rhythmicity in oscillating gene expression in young adult animals that harbor mutations that chronically disrupt sleep. A subset of these genes similarly lost rhythmicity in aged flies.

## Materials and methods

### *Drosophila* strains and culture

*Drosophila melanogaster* stocks were raised on standard media (cornmeal/yeast/molasses/agar) at 25 °C under 12-hour light/12-hour dark (LD) cycles. Wild type isogenic strain *Canton-S w* ^*1118*^ (*iso1CJ*) (Li et al., 2013a), referred to as *WT*, was used as the control for behavioral experiments and RNA-seq experiments. Sleep mutant strain *inc* ^*1*^ (Stavropoulos and Young, 2011) was a standing stock of the laboratory. Sleep mutant strain *wake* ^*D2*^ (Liu et al., 2014) was kindly provided by Dr. Mark Wu. Both mutant strains were backcrossed to the wild type strain *Canton-S w* ^*1118*^ (*iso1CJ*) for at least 5 generations.

### Sleep measurement and analysis

Locomotor activity of flies was collected using the *Drosophila* Activity Monitor (DAM) system (TriKinetics, Waltham, MA). Flies were singly housed in glass tubes containing fly culture food and assayed at 25°C under LD cycles. Activity counts were collected at 1-min bins for 5 LD cycles immediately after the loading day. DAM monitor files were processed with pySolo software (Gilestro and Cirelli, 2009). Sleep was determined as at least 5 min of inactivity. Sleep duration and sleep bouts were analyzed with pySolo software.

**FIGURE 1:**
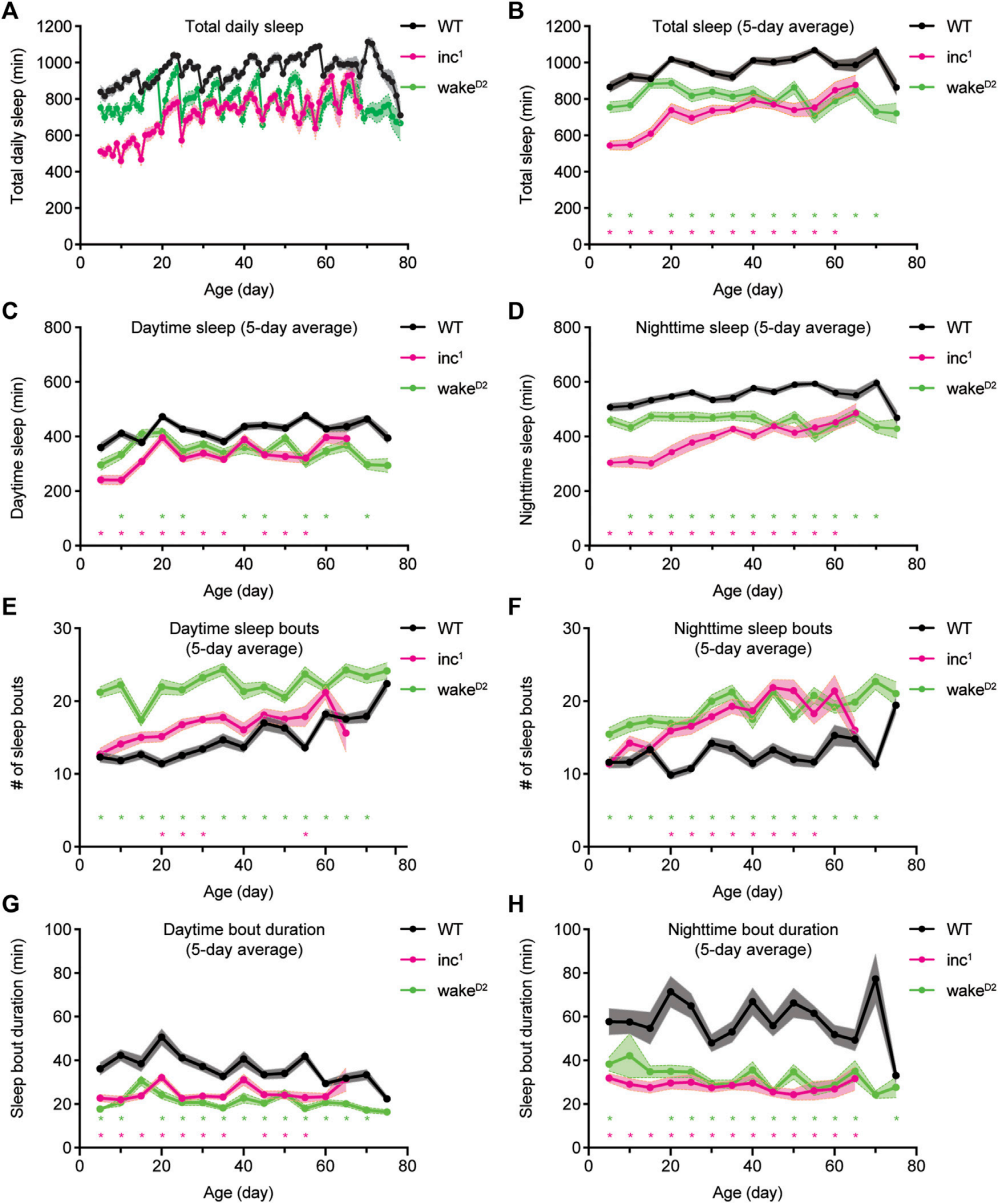
Chronic and consistent sleep loss in sleep mutants *inc* ^*1*^ and *wake* ^*D2*^. **(A)** Average total sleep per day for *WT* (black), *inc* ^*1*^ (magenta), and *wake* ^*D2*^ (green) male flies over lifetime. **(B–D)** 5-day average total sleep per day **(B)**, average daytime sleep **(C)**, and average nighttime sleep **(D)** for *WT* (black), *inc* ^*1*^ (magenta), and *wake* ^*D2*^ (green) male flies over lifetime. Each data point represents flies of corresponding age that were loaded and measured for sleep over 5 consecutive days. Data collected from different loadings are connected by solid line. **(E,F)** Average number of daytime sleep bouts **(E)** and nighttime sleep bouts **(F)** for *WT* (black), *inc* ^*1*^ (magenta), and *wake* ^*D2*^ (green) male flies over lifetime. **(G, H)** Average duration of daytime sleep bouts **(G)** and nighttime sleep bouts **(H)** for *WT* (black), *inc* ^*1*^ (magenta), and *wake* ^*D2*^ (green) male flies over lifetime. Mean ± SEM is shown. *n* = 26–32 for *WT*, n = 4–32 for *inc* ^*1*^, n = 11–32 for *wake* ^*D2*^. *p < 0.05; multiple t tests with Holm-Sidak correction against same-age *WT* flies.

For sleep measurements across the lifespan of control and mutant flies, flies were housed and aged in food bottles, with males and females housed together in each bottle. Flies were flipped every week into fresh food bottles. Batches of male flies were loaded into the DAM system every 5 days at designated ages and sleep was measured over 5 consecutive days. All flies used in Figure 1 were born around the same time and aged the same way. Each data point was collected from flies raised and aged in the same bottles.

### Longevity assay

Parent flies were raised on standard media for 2–3 days and then removed. Offspring were collected within 24 h after eclosion and were raised in LD cycles at 25°C for 2 days. Subsequently, 15 male and 15 female animals were placed into each individual food vial, and the vials were placed in LD cycles at 25 °C. Animals were transferred to new food vials every 1–3 days and the number of dead animals was recorded. dLife software was used to analyze longevity data (Linford et al., 2013). Log-rank tests were performed to determine statistical significance. Flies that escaped during transfer were included in the analysis as right-censored events.

### RNA extraction

Heads of about 200 flies of mixed sexes were collected at designated age and time. For RNA-seq experiments, four groups of samples were collected, *WT* at Day 27 as control group, *inc* ^*1*^ at Day 27, *wake* ^*D2*^ at Day 27, and *WT-old* at Day 49. Samples were collected every 4 h over 2 days of LD cycles, yielding 12 timepoints per genotype. Total RNA was extracted using TRIzol (Invitrogen) reagents and homogenized using a BeadBug microtube homogenizer (Benchmark Scientific). Samples were further extracted using chloroform and the aqueous phase containing nucleic acids was harvested. RNeasy Mini Kit (Qiagen) was used to remove DNA with DNase and further purify the samples, according to the manufacturer’s protocol.

### RNA sequencing

RNA-seq was conducted at the Genomics Resource Center of the Rockefeller University. Sequencing libraries were prepared with the Illumina TruSeq stranded mRNA LT kit. 100 ng of total RNA for each sample was used. Libraries were multiplexed and sequenced on the Illumina NextSeq 500 sequencer using high output V2 reagents and NextSeq Control Software v1.4 to generate 75bp single reads, following manufacturer’s protocol. The sequencing depth was about 25 million reads per sample. Reads were aligned to the *D. melanogaster* reference genome assembly (Release 6.13) with STAR and read counts were generated with featureCounts (dos Santos et al., 2015; Dobin et al., 2013; Liao et al., 2014). Genes with cpm values less than 1 in more than 28 samples in the entire dataset were considered as low expression and were removed from further analysis. Pearson’s correlations between samples were calculated using package in R. PCA was performed using module implemented in DESeq2 (Love et al., 2014).

### Differential gene expression analysis

DGE analysis was performed with DESeq2 (Love et al., 2014) and edgeR (Robinson et al., 2010; McCarthy et al., 2012) with recommended parameter settings for time series analysis. Specifically, likelihood ratio test (LRT) was used in DESeq2 and generalized linear model (GLM) was used in edgeR. Differentially expressed genes were selected with significance cutoffs of BH adjusted *p* < 0.05 for DESeq2 and *p* < 0.05 for edgeR. Gene ontology (GO) analysis was performed using the Database for Annotation, Visualization, and Integrated Discovery (DAVID) (Huang da et al., 2009a; Huang da et al., 2009b).

### Circadian analysis

Rhythmically expressed genes were identified using ARSER (Yang and Su, 2010), JTK cycle (Hughes et al., 2010), and GeneCycle (Wichert et al., 2004; Ahdesmaki et al., 2005; Ahdesmaki et al., 2007). For all three programs, input data was formatted as a series of two consecutive cycles. Genes whose cycling expression was deemed significant by at least two programs were considered as oscillating (Koike et al., 2012). For phase distribution, peak expression times calculated by JTK cycle were used. For change of rhythmicity analysis, highly rhythmic genes were defined as BH adjusted *p* < 0.01 and highly arrhythmic genes as BH adjusted *p* > 0.5, with BH adjusted *p* values calculated by JTK cycle.

### Statistical analysis

Statistical analysis for sleep experiments was performed using Prism 8 (GraphPad) with specific statistical tests described in figure legends. Statistical analysis for longevity experiments was performed using dLife. RNA-seq experiments were analyzed in R.

## Results

### Sleep loss throughout lifetime in sleep mutants

To achieve chronic and consistent sleep loss, we monitored the sleep behavior of sleep mutants *insomniac* ^*1*^ (*inc* ^*1*^) and *wide awake* ^*D2*^ (*wake* ^*D2*^) over their entire lifespans (Figure 1). *inc* ^*1*^ and *wake* ^*D2*^ were selected because 1) both *inc* ^*1*^ and *wake* ^*D2*^ demonstrate robust sleep loss phenotypes; 2) both *inc* and *wake* function in the brain to regulate sleep; and 3) *inc* and *wake* are involved in distinct biological pathways; 4) both mutants exhibit a reduction in lifespan, indicating suboptimal levels of sleep (Stavropoulos and Young, 2011; Liu et al., 2014) (Supplementary Figure S1). The mutants were backcrossed to the same genetic background using an isogenized wild type strain *iso1CJ* (Li et al., 2013a), hereafter referred to as *WT*. It has been reported that compared to closely related *w* ^*1118*^ strain, *WT* flies show deeper sleep especially at night (Faville et al., 2015).

To quantify sleep behavior over the entire lifespan, batches of control and mutant flies were loaded into the *Drosophila* Activity Monitor (DAM) system every 5 days at specific ages and tested for sleep over 5 consecutive days. Sleep mutants *inc* ^*1*^ and *wake* ^*D2*^ consistently showed decreased amounts of total sleep, daytime sleep, and nighttime sleep than wild type animals (Figures 1A–D). Variations in total sleep, daytime sleep, and nighttime sleep were observed within each genotype. Measurements of sleep duration mildly increased in old *WT* flies. The daily sleep time, daytime sleep, and nighttime sleep of *wake* ^*D2*^ animals fluctuated around fixed levels. However, total sleep of *inc* ^*1*^ flies increased substantially in old animals, most of which could be attributed to increased nighttime sleep (Figures 1C,D). Analysis of sleep bouts showed that over the entire course of lifelong sleep measurement, both *inc* ^*1*^ and *wake* ^*D2*^ mutant animals showed higher numbers of sleep bouts and bouts of shorter duration compared to control animals (Figures 1E–H). The improvement of sleep with age observed for *inc* ^*1*^ could be due to survivorship bias; *inc* ^*1*^ flies that managed to get better sleep throughout life might be more likely to live longer. Furthermore, *inc* ^*1*^ and *wake* ^*D2*^ animals showed significantly shortened lifespans, suggesting that the mutant flies suffered from physiological defects due to chronic sleep loss (Supplementary Figure S1). Taken together, sleep mutants *inc* ^*1*^ and *wake* ^*D2*^ offer exceptional genetic materials to study the consequences of chronic sleep loss.

### Transcriptome scale changes of rhythmic gene expression following chronic sleep loss

To study the effects of chronic sleep loss on gene oscillation, we performed transcriptome profiling using RNA-sequencing (RNA-seq) to investigate the changes on gene expression in *inc* ^*1*^ and *wake* ^*D2*^ animals. Because *inc* and *wake* have diverse physiological functions, an intersectional approach using both mutants can enrich the gene expression changes that result from chronic sleep loss.

The age for sample collection was determined based on the longevity profiles of the mutants and *WT* animals (Supplementary Figure S1B). Both male and female flies of the same genotype were housed together, as social environment has been reported to affect sleep in flies (Li et al., 2021). Within-group comparison between male flies and female flies did not show significant differences for *WT* (*p* > 0.05) or *wake* ^*D2*^ (*p* > 0.05). *inc* ^*1*^ flies displayed a moderate sex-dependent difference in survivorship (*p* < 0.05) which was more evident after Day 50 (Supplementary Figure S1B). For both males and females, same-sex comparison of sleep mutants against control animals all showed significantly reduced longevity in sleep mutants (*p* < 0.0001) (Supplementary Figure S1B). We collected heads from 4-week-old animals, before the rapid descent of their longevity curves. Samples were collected every 4 h over 2 days of 12-hour light/12-hour dark (LD) cycles, yielding 12 timepoints per genotype. RNA-seq results showed high within-group correlations (Supplementary Figure S2A). Principal component analysis (PCA) showed clear separations among genotypes (Supplementary Figure S2B).

**FIGURE 2:**
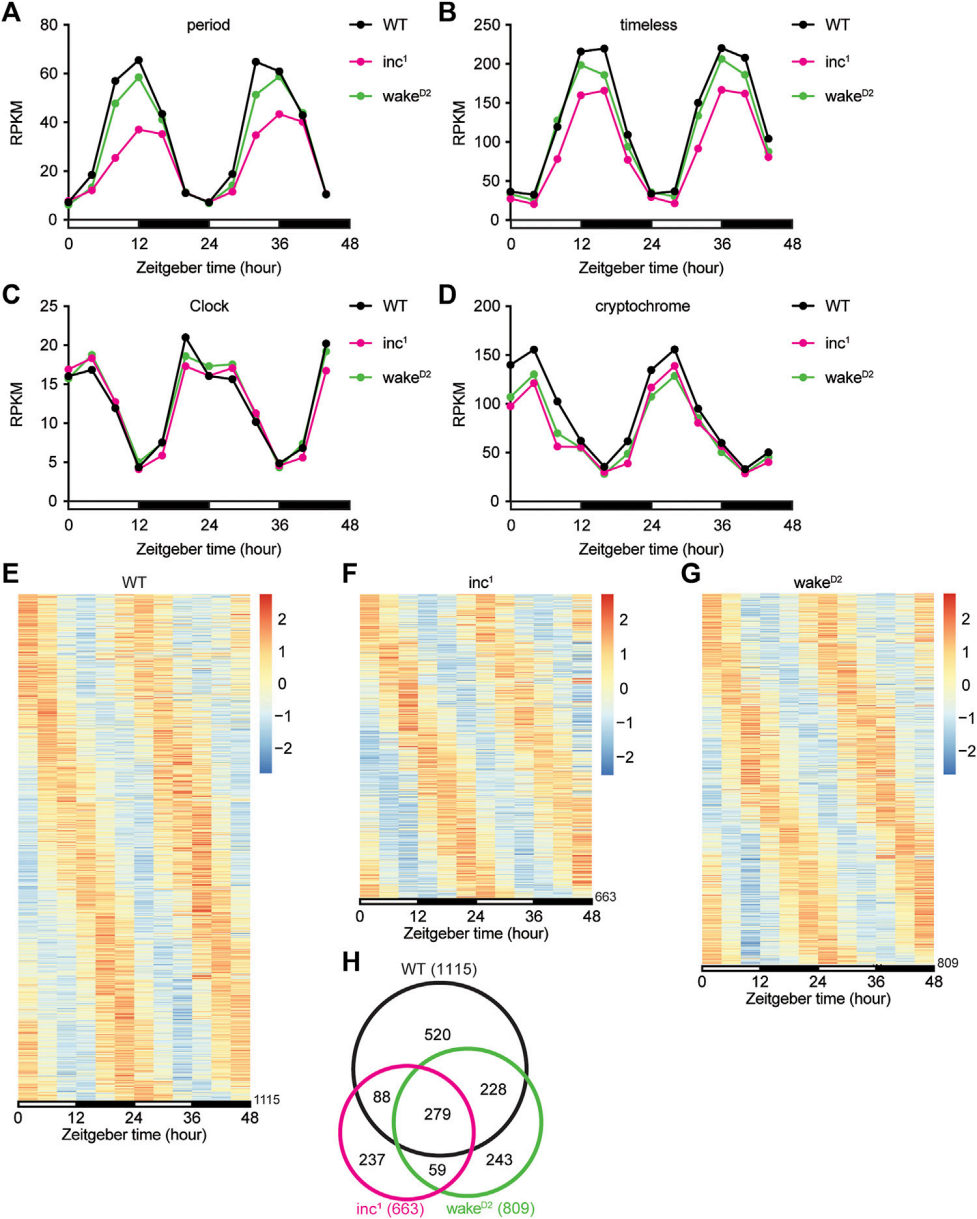
Chronic sleep loss by *inc* or *wake* mutations disrupts circadian gene oscillation. **(A–D)** Reads Per Kilobase of transcript, per Million mapped reads (RPKM) of *period* **(A)**, *timeless* **(B)**, *Clock* **(C)**, and *cryptochrome* **(D)** for *WT* (black), *inc* ^*1*^ (magenta), and *wake* ^*D2*^ (green). x axis represents two 12-hour light/12-hour dark (LD) cycles with white bars representing daytime and black bars representing nighttime. For *period*, **** *inc* ^*1*^ and n.s. *wake* ^*D2*^. For *timeless*, **** *inc* ^*1*^ and n.s. *wake* ^*D2*^. For *Clock*, n.s. *inc* ^*1*^ and *wake* ^*D2*^. For cryptochrome, **** *inc* ^*1*^ and *wake* ^*D2*^. All comparisons are against *WT*, using Benjamini-Hochberg adjusted *p* values calculated by DESeq2 for time-series analysis. ****p < 0.0001; n.s., not significant. **(E–G)** Heatmaps of rhythmic genes in *WT* **(E)**, *inc* ^*1*^ **(F)**, *wake* ^*D2*^ **(G)** groups. Each row represents one gene, and each column represents one timepoint. Expression levels are normalized to each gene itself and data are aligned according to the peak expression time calculated by JTK cycle. The blocked horizontal bars below the panel correspond to the light-dark cycle. **(H)** Intersection of rhythmic genes in *WT* (black), *inc* ^*1*^ (magenta), and *wake* ^*D2*^ (green).

We first inspected the core circadian clock genes, *period* (*per*), *timeless* (*tim*), *Clock* (*Clk*), and *cryptochrome* (*cry*) (Figures 2A–D). It was clear that the Reads Per Kilobase of transcript, per Million mapped reads (RPKM) from the RNA-seq dataset for all 4 core clock genes oscillated with a 24-hour period in all 3 groups. However, some deviations from the control group were observed (Figures 2A–D). The amplitudes of oscillation in *inc* ^*1*^ flies were significantly lower for *per* (Benjamini-Hochberg (BH) adjusted *p* < 0.0001), *tim* (BH adjusted *p* < 0.0001), and *cry* (BH adjusted *p* < 0.0001). In *wake* ^*D2*^, oscillation of *cry* was dampened compared to *WT* (BH adjusted *p* < 0.0001). These results suggested oscillation amplitudes of core clock genes were altered in sleep mutants, albeit the core clock genes remain robustly rhythmic.

Oscillating genes in whole transcriptome were selected using a combinatorial approach of multiple bioinformatic algorithms, including ARSER (Yang and Su, 2010), JTK cycle (Hughes et al., 2010), and GeneCycle (Wichert et al., 2004; Ahdesmaki et al., 2005; Ahdesmaki et al., 2007). Genes whose cycling expression was deemed significant by at least two programs were considered as oscillating (Koike et al., 2012). Using this criterion, we found that 1115 genes exhibited robust oscillation in the control *WT* flies (Figures 2E, Supplementary File S1), which was comparable to previous studies (Hughes et al., 2012; Kuintzle et al., 2017). We performed the same analysis to identify oscillating transcripts in sleep mutants *inc* ^*1*^ and *wake* ^*D2*^ (Figures 2F,G). Compared to control *WT* flies, a smaller number of genes were identified as rhythmic in the two sleep mutants, 663 in *inc* ^*1*^ (Supplementary File S2) and 809 in *wake* ^*D2*^ (Supplementary File S3), suggesting a strong disruption of transcriptome-level gene oscillation compared to *WT*. About 50% of the oscillating genes identified in *inc* ^*1*^ and *wake* ^*D2*^ mutants were present in the set of oscillating genes identified in *WT* (Figure 2H). The intersection of oscillating genes in all 3 groups resulted in a set of 279 genes (Khan and Mathelier, 2017), suggesting that a significant portion of *WT* program of rhythmic gene expression is not abolished after chronic sleep loss (Figure 2H).

Surprisingly, in comparison to *WT* flies, a number of genes gained rhythmic patterns of expression in *inc* ^*1*^ and *wake* ^*D2*^ mutants. However, as described in more detail below, these newly rhythmic genes were less often common to both sleep mutants (Figure 2H).

Since the numbers of oscillating genes dramatically decreased in sleep mutants, we evaluated the impact on the phase distributions of rhythmic gene expression in the two sleep mutants. As demonstrated by the circular histograms representing the distribution of peak expression time of oscillating genes, phase distributions among the reduced sets of oscillating genes resembled those of *WT* (Supplementary Figure S3).

### Alteration in rhythmicity induced by chronic sleep loss

**FIGURE 3:**
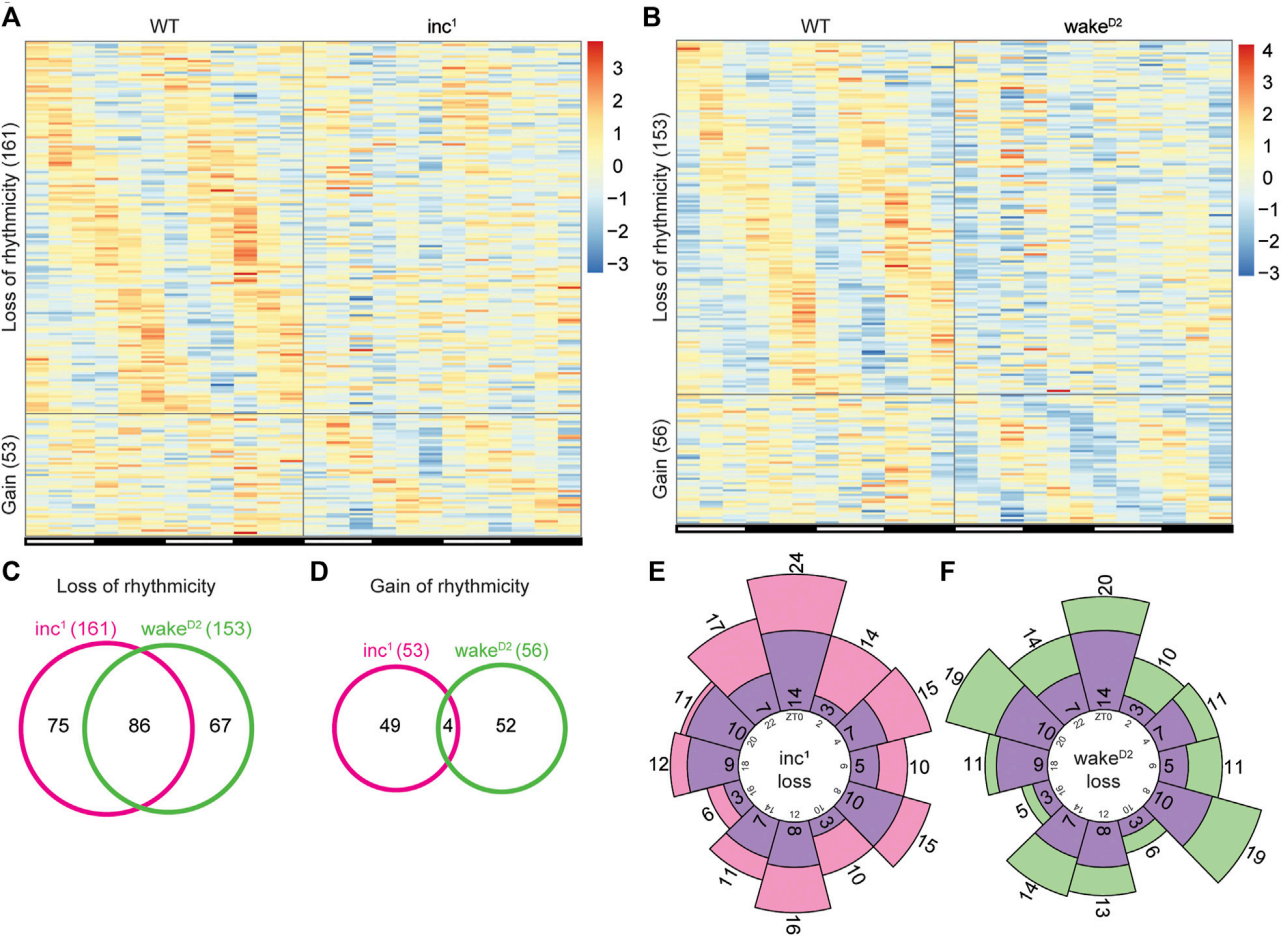
Identification of rhythmicity changes in chronically sleep deprived flies. **(A,B)** Genes with rhythmicity change in *inc* ^*1*^ **(A)** and *wake* ^*D2*^ **(B)** flies. “Loss of rhythmicity” represents genes that are highly rhythmic in *WT* and highly arrhythmic in the experimental group, and *vice versa* for “Gain”. Highly rhythmic genes are defined as Benjamini-Hochberg adjusted *p* < 0.01 and high arrhythmic genes as Benjamini-Hochberg adjusted *p* > 0.5, calculated by JTK cycle. Each row represents one gene, and each column represents one timepoint. Expression levels are normalized to each gene itself and data are aligned according to the peak expression time in the highly rhythmic group calculated by JTK cycle. The blocked horizontal bars below the panel correspond to the light-dark cycle. **(C,D)** Intersection of loss of rhythmicity genes **(C)** and gain of rhythmicity genes **(D)** in *inc* ^*1*^ (magenta) and *wake* ^*D2*^ (green). **(E,F)** Circular histogram for the distribution of *WT* peak expression time for loss of rhythmicity genes in *inc* ^*1*^ **(E)** and *wake* ^*D2*^ **(F)**. Purple bars represent the distribution of *WT* peak expression time for the 86 intersected genes in **(C)**. Peak expression times are calculated from the *WT* samples using JTK cycle. Numbers in the inner circle represent Zeitgeber time (ZT) and numbers in or adjacent to the bars represent number of genes in the corresponding bar.

To further investigate alterations in rhythmicity after chronic sleep loss, we compared the list of rhythmic genes in each sleep mutant with that of the *WT* control group, focusing on genes that were found to be highly rhythmic in one group but highly arrhythmic in the other. Interestingly, when the two sleep mutants were individually compared with *WT*, the scale of rhythmicity change was comparable (Figure 3A,B). Compared to *WT*, 161 and 153 genes lost rhythmicity in *inc* ^*1*^ and *wake* ^*D2*^, respectively; the gain of rhythmicity groups were much smaller, 53 genes for *inc* ^*1*^ and 56 genes for *wake* ^*D2*^ (Supplementary File S4,S5). These results confirmed that in both sleep mutants, circadian gene oscillation was affected.

Furthermore, more than 50% (86 genes) of the loss of rhythmicity genes were shared between *inc* ^*1*^ and *wake* ^*D2*^, but less than 10% (4 genes) of the genes that gained rhythmicity were shared between *inc* ^*1*^ and *wake* ^*D2*^ (Figure 3C,D). These data suggested that the majority of the rhythmicity loss might be linked to chronic sleep disruption, while the gain of rhythmic gene activity appeared to be independent of sleep phenotype.

To assess whether loss of rhythmicity was enriched for a specific timepoint(s), we examined the phase distribution of loss of rhythmicity genes using peak expression time in the *WT* group (Figures 3E,F). We did not observe timepoint specific enrichment of the peak expression time. Furthermore, there was no significant difference between the phase distributions for the loss of rhythmicity genes in *inc* ^*1*^ and *wake* ^*D2*^ (*p* > 0.05, Chi-square test), nor between the sleep mutants and the 86 intersected genes (*p* > 0.05, Chi-square test).

**FIGURE 4:**
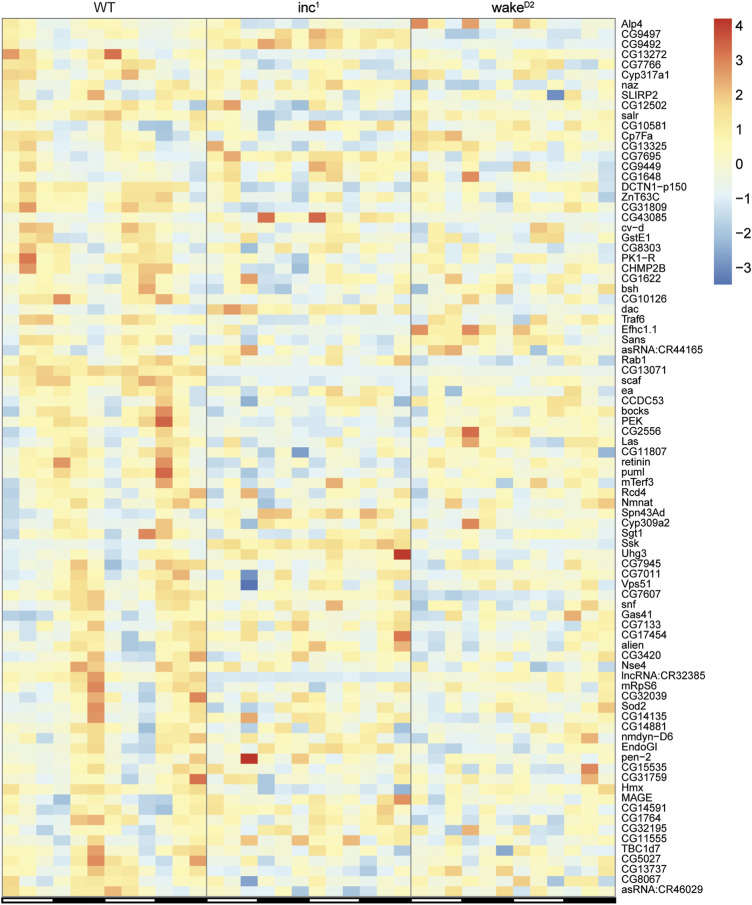
Expression of the loss of rhythmicity genes in chronically sleep deprived flies. Each row represents one gene, and each column represents one timepoint. Expression levels are normalized to each gene itself and data are aligned according to the peak expression time in *WT* group calculated by JTK cycle. The blocked horizontal bars below the panel correspond to the light-dark cycle.

The expression patterns of the 86 genes that lost rhythmicity in both sleep mutants are shown in Figure 4. Although gene ontology analysis using the Database for Annotation, Visualization, and Integrated Discovery (DAVID) (Huang da et al., 2009a; Huang da et al., 2009b) did not reveal significantly enriched biological processes, these genes provided valuable information regarding the impact of chronic sleep loss on gene oscillation. *Presenilin enhancer* (*pen-2*) is believed to be required for Notch pathway signaling and activity of γ-secretase, a critical player in the development of Alzheimer’s disease (Francis et al., 2002). *Pyrokinin 1 receptor* (*PK1-R*) encodes a G-protein coupled receptor (GPCR) and is thought to regulate the production and release of insulin (Alfa et al., 2015). *TNF-receptor-associated factor 6* (*Traf6*) and *easter* (*ea*) are both involved in Toll related receptor signaling, an essential component of innate immunity (Cha et al., 2003; Jang et al., 2006). Overexpression of reactive oxygen species regulator *Superoxide dismutase 2* (*Sod2*) has been shown to extend lifespan in *Drosophila* (Curtis et al., 2007). *Non-SMC element 4* (*Nse4*) and *MAGE* encode components of the SMC5/6 protein complex, which is crucial for homologous DNA recombination-based DNA repair (Gaudet et al., 2011; Li et al., 2013b). Abnormalities in the rhythmicity of these genes might translate into severe physiological defects affecting lifespan, which remains to be investigated in closer detail.

### Differential gene expression in chronically sleep deprived animals

**FIGURE 5:**
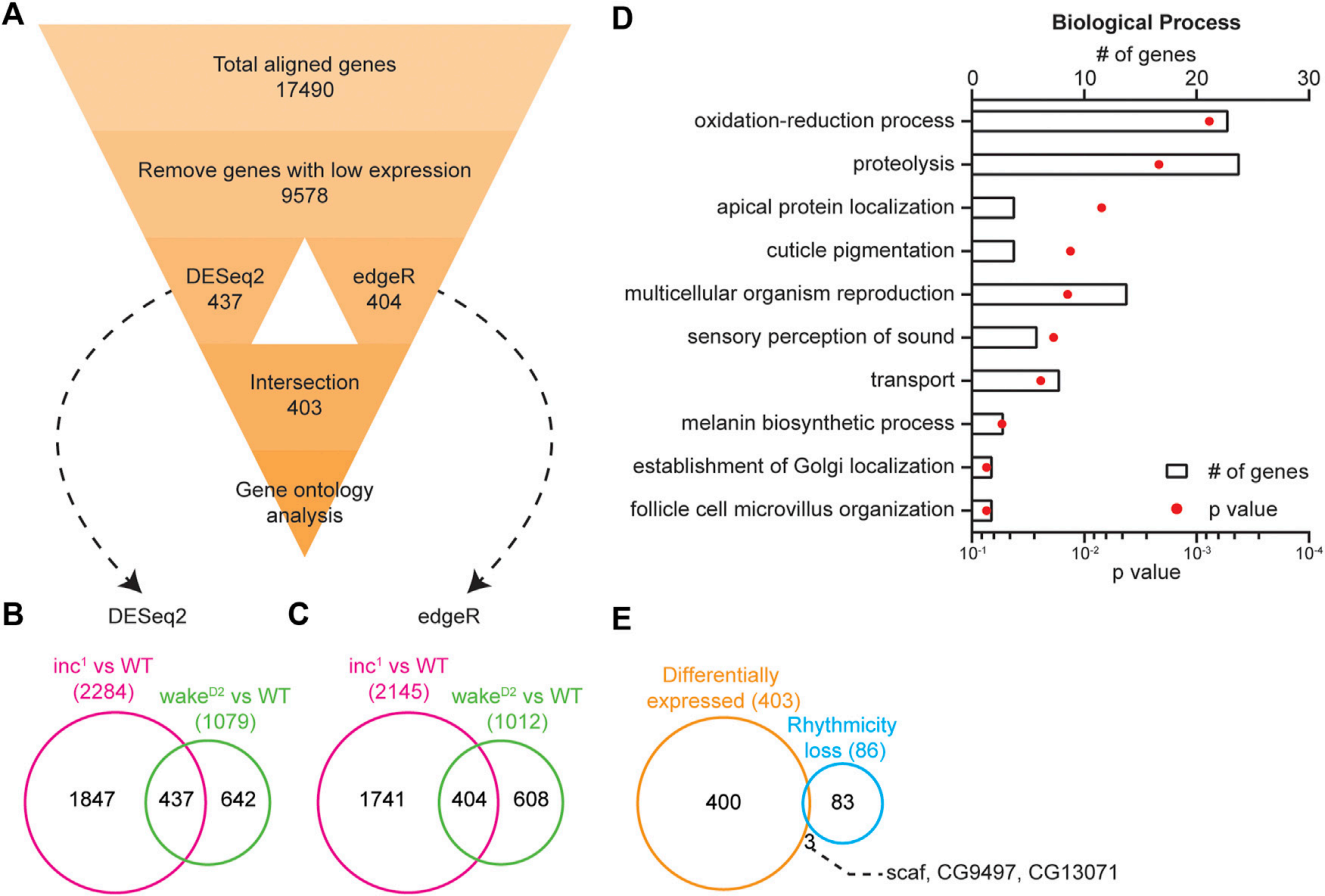
Loss of rhythmicity is not caused by differential expression. **(A)** Flowchart showing the pipeline for differential gene expression (DGE) analysis. Number in each box represents number of genes. **(B)** Intersection of differentially expressed genes identified by DESeq2 (Benjamini-Hochberg adjusted *p* < 0.05) in *inc* ^*1*^ (magenta) and *wake* ^*D2*^ (green). **(C)** Intersection of differentially expressed genes identified by edgeR (*p* < 0.05) in *inc* ^*1*^ (magenta) and *wake* ^*D2*^ (green). **(D)** Enriched biological processes revealed by gene ontology analysis using 403 differentially expressed genes. Top 10 enriched biological pathways are shown. Bars represent number of genes and dots represent *p*-value. **(E)** Intersection of differentially expressed genes (orange) and loss of rhythmicity genes (cyan) in both *inc* ^*1*^ and *wake* ^*D2*^ animals. The 3 intersected genes are listed.

A previous microarray study identified many transcripts with altered gene expression level after 1-week of sleep deprivation in rats using the disk-over-water deprivation method (Cirelli et al., 2006). To understand whether the losses of rhythmicity observed in *inc* ^*1*^ and *wake* ^*D2*^ were due to similar changes in average expression level, we performed differential gene expression (DGE) analysis to identify genes that exhibited significantly elevated or dampened expression relative to their expression levels in control *WT* flies. Figure 5A illustrates the workflow of the DGE analysis (see Materials and methods). Two algorithms, DESeq2 (Love et al., 2014) and edgeR (Robinson et al., 2010; McCarthy et al., 2012), were used to identify genes whose expression levels in the experimental group were significantly different from those of the control group independent of timepoints. When compared individually against *WT* samples, the results from the two sleep mutants differed substantially (Figure 5B,C). Over 2,000 genes were found to be differentially expressed in *inc* ^*1*^, while the algorithms identified about 1,000 differentially expressed genes in *wake* ^*D2*^.

The results from both comparisons were subsequently intersected. DESeq2 detected 437 genes and edgeR 404 genes. Further intersection of the two algorithms resulted in a list consisting of 403 genes (Supplementary File S6). These analyses demonstrated the power of the intersection approach with two sleep mutants, whereas the choice of bioinformatic programs for DGE did not impose a great influence on the results. With the 403 differentially expressed genes, we conducted gene ontology analysis to examine the enriched biological processes (Huang da et al., 2009a; Huang da et al., 2009b). The top enriched biological processes include oxidation-reduction process, proteolysis, apical protein localization, cuticle pigmentation, and multicellular organism reproduction (Figure 5D).

The list of differentially expressed genes showed little intersection with that of the loss of rhythmicity genes (Figure 5E). Only 3 genes, *scarface (scaf)*, *CG9497*, and *CG13071*, were found to be present in both lists, indicating that the changes in circadian oscillation in sleep mutants were not due to changes in timepoint averaged expression levels.

### Shared rhythmicity changes across chronically sleep deprived flies and old flies

**FIGURE 6:**
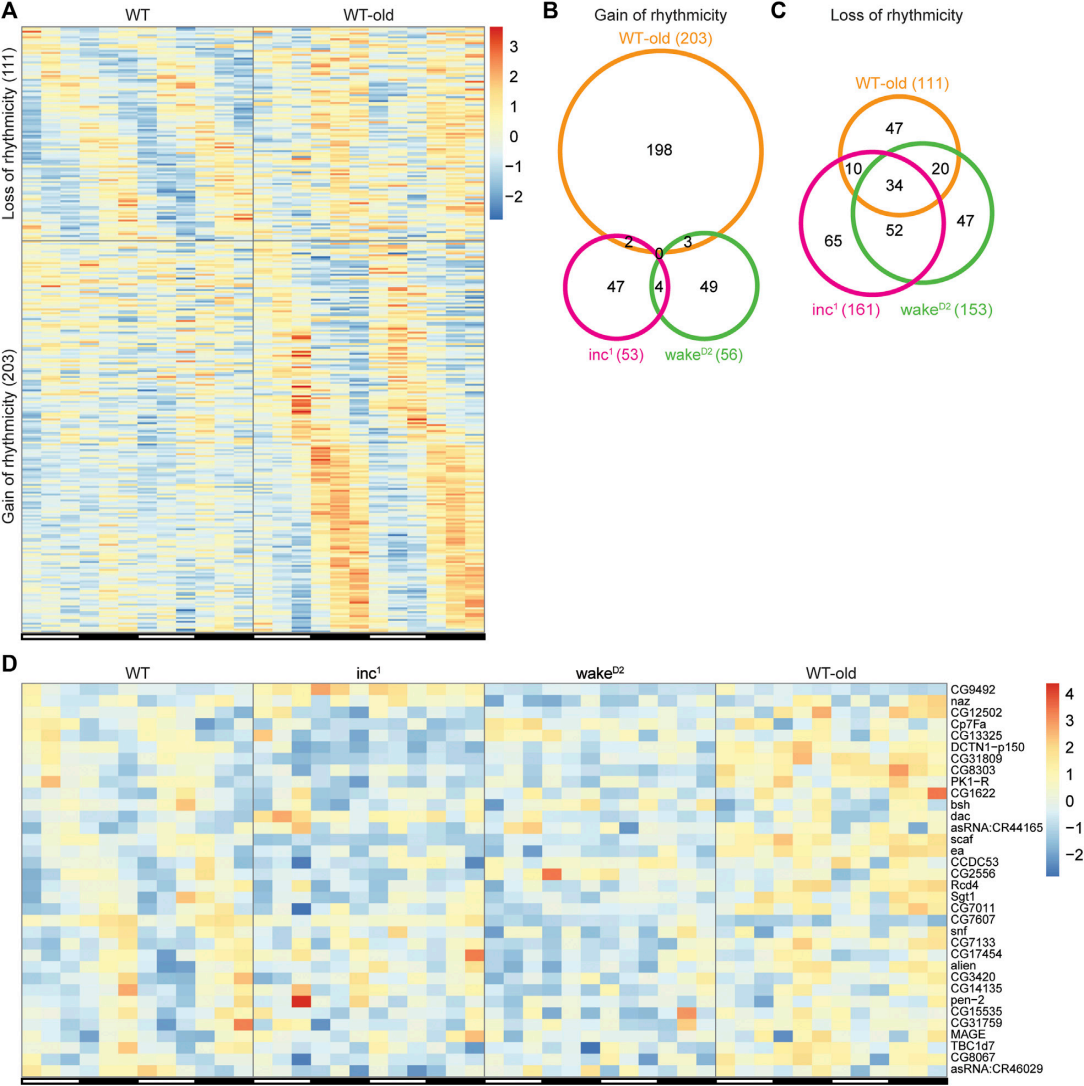
Shared rhythmicity changes across chronically sleep deprived flies and old flies. **(A)** Genes with rhythmicity change in *WT-old*. Each row represents one gene, and each column represents one timepoint. Expression levels are normalized to each gene itself and data are aligned according to the peak expression time in the highly rhythmic group calculated by JTK cycle. The blocked horizontal bars below the panel correspond to the light-dark cycle. **(B,C)** Intersection of gain of rhythmicity genes **(B)** and loss of rhythmicity genes **(C)** in *inc* ^*1*^ (magenta), *wake* ^*D2*^ (green), and *WT-old* (orange). **(D)** Heatmap of the 34 intersected genes in **(C)**. Each row represents one gene, and each column represents one timepoint. Expression levels are normalized to each gene itself and data are aligned according to the peak expression time in *WT* group calculated by JTK cycle. The blocked horizontal bars below the panel correspond to the light-dark cycle.

Alterations in circadian gene rhythmicity have recently been reported in aged flies (Kuintzle et al., 2017). To examine the possibility that genes with altered rhythmicity in sleep mutants exhibit rhythmicity changes in old flies, we performed transcriptome profiling using heads collected from 7-week-old *WT* flies, referred to as *WT-old*. The core clock genes remain robustly oscillating in *WT-old*, suggesting the integrity of the master clock in aged flies (Supplementary Figure S4). However, we discovered 111 genes that lost rhythmicity and 203 genes that gained rhythmicity in *WT-old* compared to control animals (Figure 6A, Supplementary File S7). The higher abundance of gain of rhythmicity genes in old flies was consistent with findings reported by Kuintzle, et al. (Kuintzle et al., 2017). Additionally, 20 out of 111 loss-of-rhythmicity genes and 61 out of 203 gain-of-rhythmic genes were found to be differentially expressed in *WT-old* compared to *WT* (Supplementary File S8).

The results from both sleep mutants and *WT-old* were intersected to detect genes with similar rhythmicity alterations among experimental groups. None of the gain of rhythmicity genes overlapped across the three experimental groups (Figure 6B), suggesting that the *de novo* gain of rhythmicity is context specific. Although there were fewer genes in the loss of rhythmicity group than genes in the gain of rhythmicity group in *WT-old*, the intersection of genes with rhythmicity loss across the experimental groups yielded a fairly large subset of overlapping genes (34 intersecting genes; Figure 6C,D, Supplementary File S9). Out of the 34 genes that lost rhythmicity in all three experimental groups, only 1 gene, *scaf*, was found to be differentially expressed in all three groups, further supporting the conclusion that changes in circadian oscillation were not due to changes in timepoint averaged expression levels (Supplementary Figure S5)). These results indicate that the disruption of circadian gene oscillation in sleep mutants shares some biological pathways with aging or longevity regulation.

## Discussion

Using sleep mutants *inc* ^*1*^ and *wake* ^*D2*^ as models for chronic sleep loss, we observed significant changes in circadian gene oscillation. Rhythmicity changes in sleep mutants *inc* ^*1*^ and *wake* ^*D2*^ were predominantly due to loss of rhythmicity, rather than changes in average expression level. Although we observed gain of rhythmicity in some genes, we did not identify any overlapping pathways or gene sets across both sleep mutants, suggesting that such changes are more likely to be related to the molecular function of *inc* or *wake*, rather than chronic sleep loss. Most interestingly, more than 50% of the genes with altered rhythmicity overlapped between the two sleep mutants. The circadian clock is believed to be intact in *inc* mutants at least within days after eclosion (Stavropoulos and Young, 2011). WAKE proteins have been shown to be rhythmically expressed in clock neurons and act downstream of CLK to regulate timing of sleep onset (Liu et al., 2014). However, we observed that the oscillation amplitudes of the components of the core circadian clock were dampened, which is likely to contribute to the disruption of circadian clock-controlled gene oscillation observed in *inc* ^*1*^ and *wake* ^*D2*^. It was previously reported that a short sleep deprivation is sufficient to perturb the core molecular circadian clock in the mouse brain even long after the mice resumed normal sleep, consistent with our data that chronic sleep deprivation dampened the core molecular clock and circadian rhythmicity profoundly (Hor et al., 2019). Furthermore, we showed that the loss of rhythmicity in *inc* ^*1*^ and *wake* ^*D2*^ was not due to changes in average expression levels of the affected genes. Since sleep-wake cycle has been shown to regulate gene oscillation independent of core molecular clock, it remains to be investigated whether the change of rhythmicity was the result of dampened oscillation of core clock genes or a direct effect of chronic sleep disruption (Maret et al., 2007). The collection of genes that lost rhythmic expression in this study of sleep mutants provides promising material for such investigations.

Large scale changes in circadian gene oscillation have been reported in aged flies (Kuintzle et al., 2017), thus we asked whether aging and chronic sleep-loss have an impact on a common set of rhythmically expressed genes. *De novo* gain of rhythmicity in aged flies was confirmed in our results. However, we observed no gene in the gain-of-rhythmicity groups that was common to *inc* ^*1*^, *wake* ^*D2*^, and old flies. In contrast, 34 genes were shared in the loss-of-rhythmicity groups by such a comparison. These results suggest that the mechanisms underlying the observed gain-of-rhythmicity are likely to be independent of the sleep phenotypes of the mutants. In contrast, certain mechanisms for loss of rhythmicity are likely promoted by both chronic sleep loss and aging.

## Data Availability

The datasets presented in this study can be found in online repositories. The RNA-Seq data generated in this study have been deposited in NCBI’s Gene Expression Omnibus (GEO) under accession code GSE148136. The raw sequence data are available for samples of all time points except for WT-ZT28 and WT-ZT40. The counts data generated using STAR and featureCounts for all RNA-seq samples are available. The code used for RNA-seq analysis and intermediary results are available at https://github.com/wangzikun329/Wang2022. RNA-seq expression plots for all genes are available at https://zikunwang.shinyapps.io/rna_seq_gene_plot/.

## Supplementary material

The Supplementary Material for this article can be found online at: https://www.frontiersin.org/articles/10.3389/fphys.2022.1048751/full#supplementary-material

Supplementary Figure S1 Longevity phenotypes of sleep mutants *inc^1^* and *wake^D2^*. **(A)** Longevity of *WT* (black), *inc^1^* (magenta), and *wake^D2^* (green) male flies. *n* = 145−150 for all groups. **** *p* < 0.0001, log-rank test against *WT* control animals. **(B)** Longevity of *WT* (black), *inc^1^* (magenta), and wakeD2 (green) male (dot) and female (triangle) flies. n = 140−150 for all groups. For log-rank test between male and female of the same genotype, *p* > 0.05 for *WT* and *wake^D2^*, *p* < 0.05 for *inc^1^*. For cross genotype log-rank test, *p* < 0.0001 for *inc^1^* and *wake^D2^* against *WT* for both males and females.

Supplementary Figure S2 Quality check for RNA sequencing results. **(A)** Heatmap showing gene expression Pearson’s correlation for all RNA-seq samples. **(B)** Principal component analysis for all RNA-seq samples.

Supplementary Figure S3 Distribution of peak expression time for oscillating genes. **(A–C)** Circular histogram for the distribution of peak expression time for rhythmic genes in *WT* **(A)**, *inc^1^* **(B)**, and *wake^D2^* **(C)**. Peak expression times are calculated using JTK cycle. Numbers in the inner circle represent Zeitgeber time (ZT) and numbers in the bars represent number of genes in the corresponding bar. Total number of oscillating genes are labeled in parentheses.

Supplementary Figure S4 Core circadian clock genes maintain robust rhythmicity in aged WT flies. **(A–D)** Reads Per Kilobase of transcript, per Million mapped reads (RPKM) of period **(A)**, timeless **(B)**, Clock **(C)**, and cryptochrome **(D)** for *WT* (black) and *WT-old* (orange). x axis represents two 12-hour light/12-hour dark (LD) cycles with white bars representing daytime and black bars representing nighttime. No significance was observed between *WT* and *WT-old*, as determined by Benjamini-Hochberg adjusted *p* values calculated by DESeq2 for time-series analysis.

Supplementary Figure S5 Identification of differentially expressed genes across chronically sleep deprived flies and old flies. **(A)** Flowchart showing the pipeline for differential gene expression (DGE) analysis. Number in each box represents number of genes. **(B)** Intersection of differentially expressed genes identified by DESeq2 (Benjamini-Hochberg adjusted *p* < 0.05) in inc1 (magenta), *wake^D2^* (green), and *WT-old* (orange). **(C)** Intersection of differentially expressed genes identified by edgeR (*p* < 0.05) in *inc^1^* (magenta), *wake^D2^* (green), and *WT-old* (orange). **(D)** Enriched biological processes revealed by gene ontology analysis using 114 differentially expressed genes. Bars represent number of genes and dots represent *p* value. **(E)** Intersection of differentially expressed genes (purple) and loss of rhythmicity genes (blue) across *inc^1^*, *wake^D2^*, and *WT-old* flies. The intersected gene is labeled.

Supplementary file S1 Oscillating genes in 4-week-old wild type flies with results calculated using JTK cycle.

Supplementary file S2 Oscillating genes in 4-week-old *inc^1^* flies with results calculated using JTK cycle.

Supplementary file S3 Oscillating genes in 4-week-old *wake^D2^* flies with results calculated using JTK cycle.

Supplementary file S4 Genes with rhythmicity change in *inc^1^* flies.

Supplementary file S5 Genes with rhythmicity change in *wake^D2^* flies.

Supplementary file S6 Intersection of differentially expressed genes in *inc^1^* and *wake^D2^* with results calculated using DESeq2 and edgeR.

Supplementary file S7 Genes with rhythmicity change in *WT-old* flies.

Supplementary file S8 Differentially expressed genes in *WT-old* with results calculated using DESeq2 and edgeR.

Supplementary file S9 ntersection of genes with rhythmicity change in *inc^1^*, *wake^D2^*, and *WT-old*.
