# Supplementary figures and images for: Chronic sleep loss disrupts rhythmic gene expression in Drosophila
Source: Front Physiol. 2022 Nov 18;13:1048751. doi: 10.3389/fphys.2022.1048751 (PMC9716074; doi:10.3389/fphys.2022.1048751)

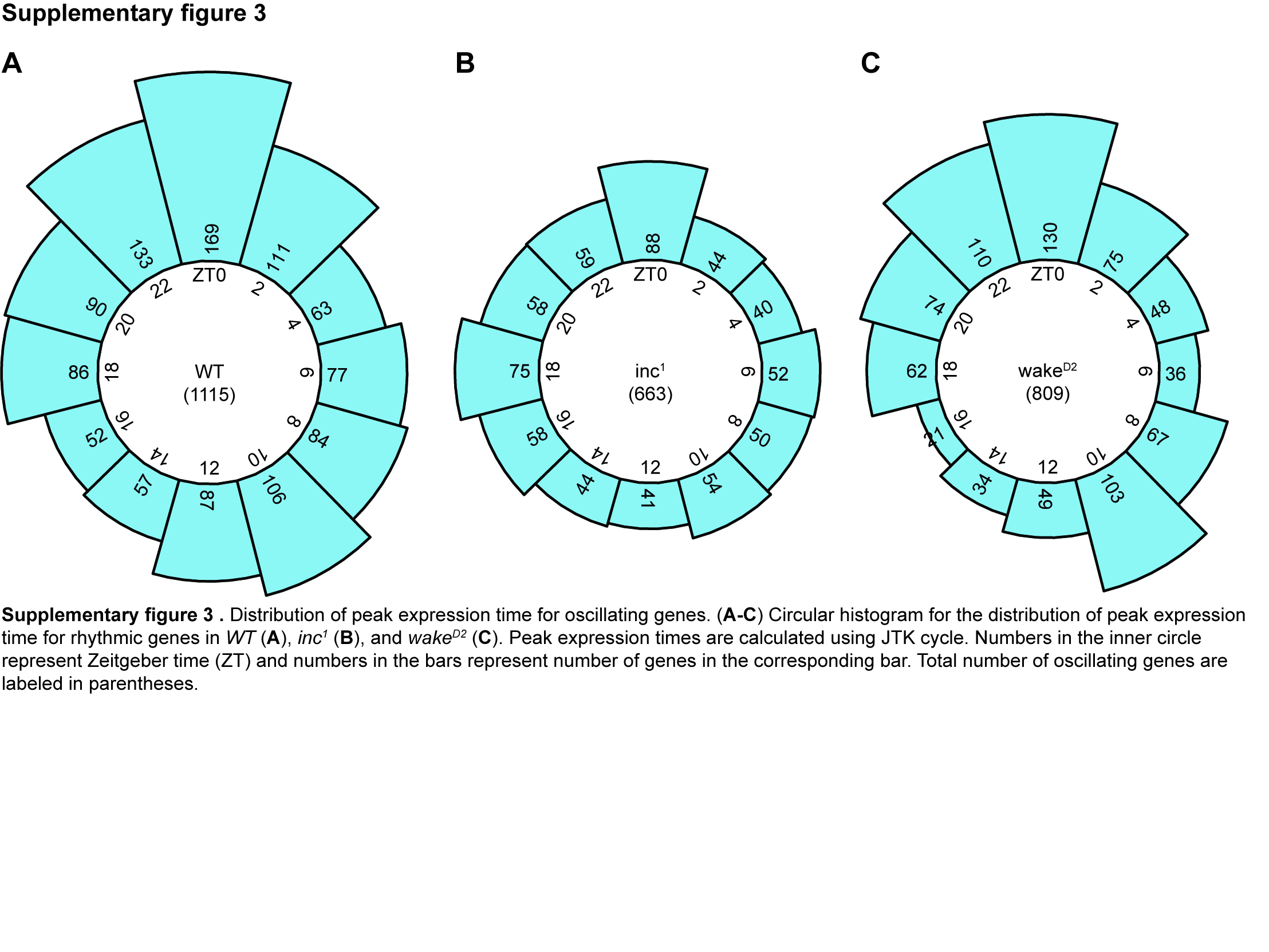

Supplement: Supplementary file 4 [file Image3.TIF]

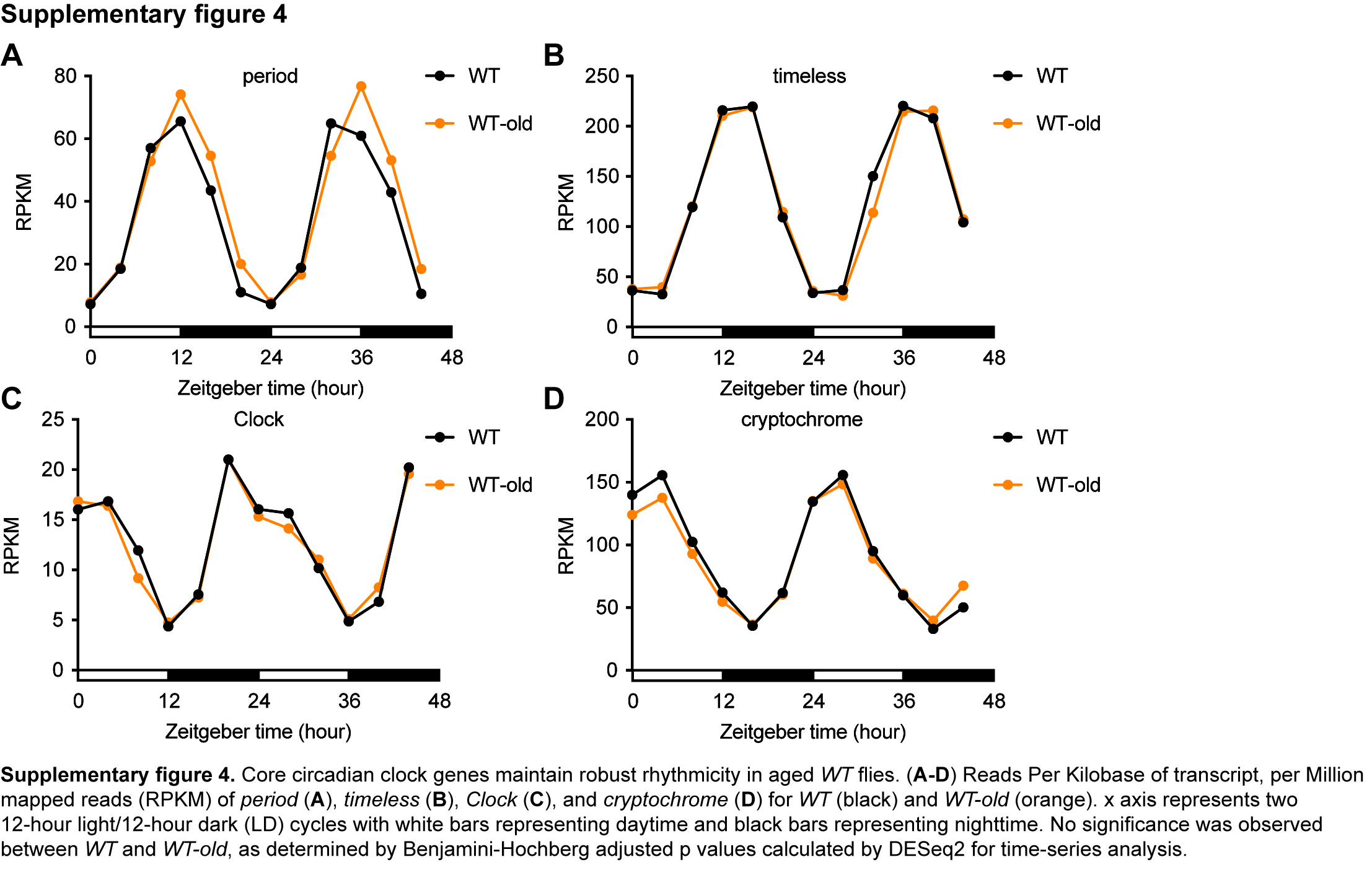

Supplement: Supplementary file 5 [file Image4.TIF]

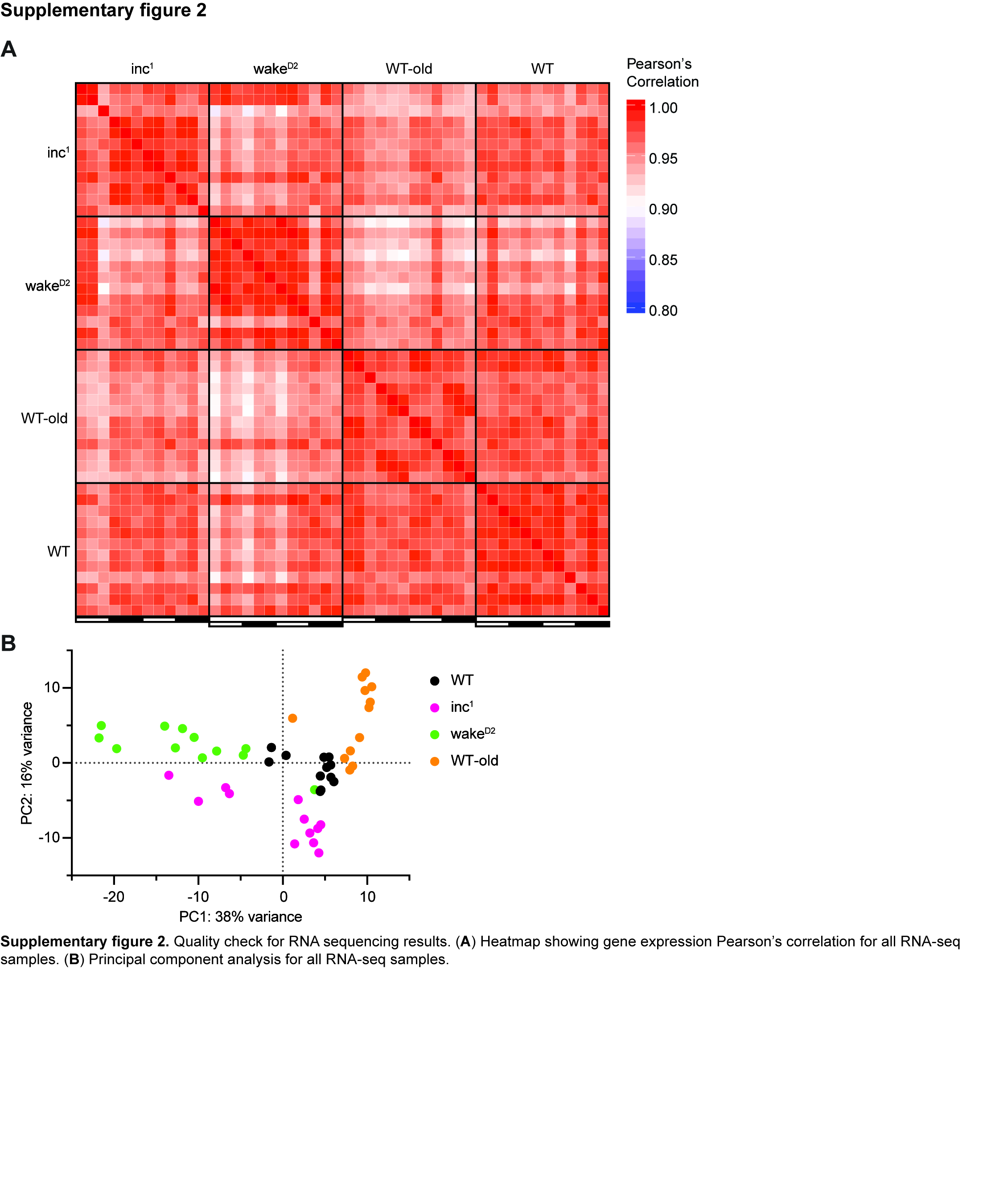

Supplement: Supplementary file 6 [file Image2.TIF]

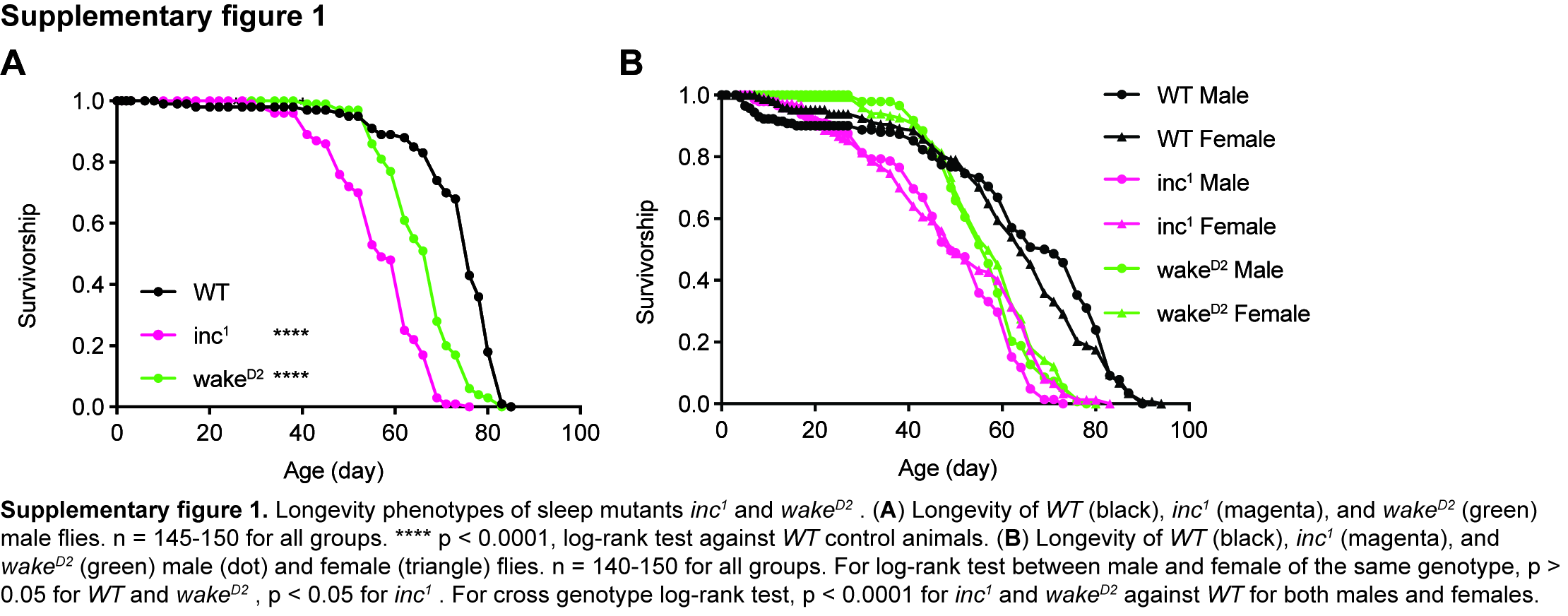

Supplement: Supplementary file 7 [file Image1.TIF]

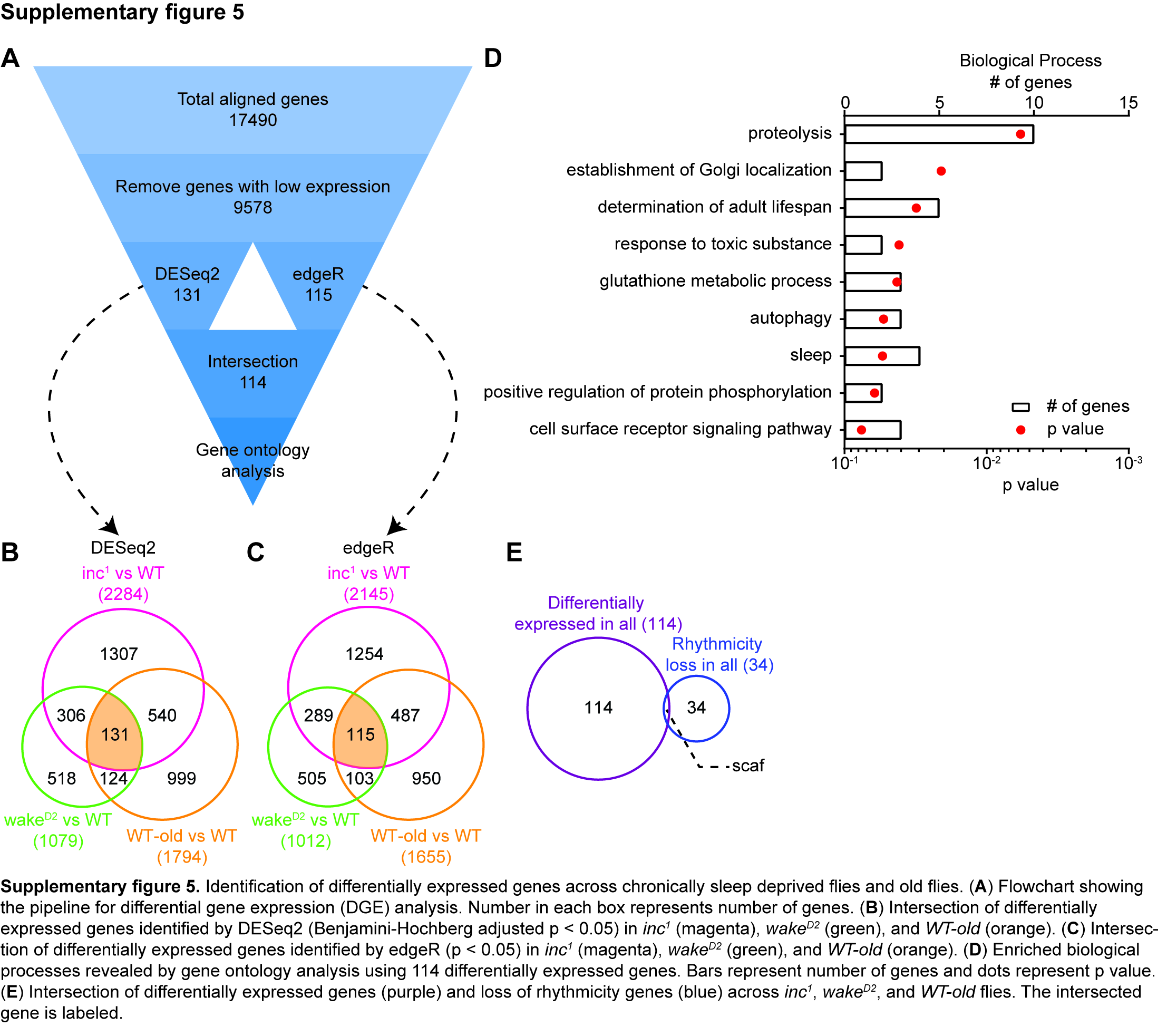

Supplement: Supplementary file 11 [file Image5.TIF]
